# Relationship between job stress and functional dyspepsia in display manufacturing sector workers: a cross-sectional study

**DOI:** 10.1186/s40557-018-0274-4

**Published:** 2018-10-19

**Authors:** Younghyeon Nam, Soon-Chan Kwon, Yong-Jin Lee, Eun-Chul Jang, Seung-hwan Ahn

**Affiliations:** 1 Department of Occupational & Environmental Medicine, Soonchunhyang University Cheonan Hospital, 31, Suncheonhyang 6-gil, Dongnam-gu, Cheonan-si, Chungcheongnam-do 31151 Republic of Korea; 2 Environmental Health Center for Asbestos, Soonchunhyang University Cheonan Hospital, 67, Suncheonhyang 3-gil, Dongnam-gu, Cheonan-si, Chungcheongnam-do 31151 Republic of Korea

**Keywords:** Functional gastrointestinal disorder, Job stress, Manufacturing industry

## Abstract

**Background:**

Job stress has been reported as a risk factor of psychological changes, which have been shown to be related to gastrointestinal diseases and symptoms such as functional dyspepsia. However, few studies have assessed the relationship between job stress and functional dyspepsia. Therefore, we investigated the relationship between job stress and functional dyspepsia in South Korea.

**Methods:**

This study was conducted between May 23 and July 6, 2016 and included 901 workers in the display manufacturing sector. Subjects completed self-reported questionnaires, regarding Korean Occupational Stress Scale (KOSS), functional dyspepsia, Insomnia Severity Index-K, and health-related behaviors and job characteristics. Subjects were divided into functional dyspepsia-positive and -negative groups based on the Rome III criteria. The KOSS high-risk group was defined as subjects with KOSS score above the 75 percentile of KOSS reference value. Multiple logistic regression analysis was performed to investigate the association between job stress and functional dyspepsia.

**Results:**

In women, the risk of functional dyspepsia was significantly higher in the high-risk groups of the following KOSS subcategories in unadjusted model: job demand (OR 3.282, 95% CI 1.181–9.126), and occupational climate (OR 2.665, 95% CI 1.041–6.823). Even in adjusted model, the risk was significantly higher in the high-risk groups of the following KOSS subcategories: job demand (OR 3.123, 95% CI 1.036–9.416) and occupational climate (OR 3.304, 95% CI 1.198–9.115). In men, the risk of functional dyspepsia was not significant in all KOSS subcategories.

**Conclusions:**

This study showed that job demand and occupational climates were associated with functional dyspepsia in female display manufacturing sector workers. Therefore, both clinical and mental health approaches should be used in the management of functional dyspepsia in women.

## Background

Functional gastrointestinal disorders are common disorders characterized by persistent and recurring gastrointestinal symptoms. The two most common functional gastrointestinal disorders are irritable bowel syndrome (IBS), and functional dyspepsia [1, 2]. Functional dyspepsia is defined as a condition with upper abdominal symptoms, such as postprandial fullness, early satiation, epigastric pain, and epigastric burning, and it occurs in the absence of organic disease that might explain their occurrence [1, 2]. Functional dyspepsia is a common disease in the general population. Its prevalence rate was reported as 7–45% globally and 8–46% in South Korea [3]. Functional dyspepsia is a multifactorial disease that can be caused by various factors such as gastric motility abnormality and visceral hypersensitivity, infection, and genetics; however, psychosocial factors are also known to be major causes [4]. A study conducted in Japan showed that patients with functional dyspepsia had a higher psychosocial factor score than the general population, and major anxiety was significantly associated with functional dyspepsia and postprandial distress syndrome [4]. Past studies suggested that non-ulcer dyspepsia had a significant relationship with depression and anxiety [5], and anxiety disorder was strongly associated with gastrointestinal symptoms [6].

Job stress can be defined as “the harmful physical and emotional responses, which occurs when the requirements of the job do not match the capabilities, resources, or needs of the worker” [7]. Job stress can lead to poor health and even injury [7]. According to a study conducted in Korea, of 6977 workers in 245 companies, 22% were classified as high-risk groups (using the Psychosocial Well-being Index), and this was reported as being associated with job stress factors [8]. Stresses have been reported to have a variety of mental health effects, ranging from mild subjective symptoms to overt psychiatric disease with significant impairment of functioning. Commonly reported symptoms are anxiety, tension, anger, irritability, poor concentration, apathy, and depression [9]. A British study showed that these stresses can increase the risk of depressive disorder and generalized anxiety disorder [10]. A Korean study showed that job stress has a positive correlation with depression, anxiety, and stress symptoms [11]. In addition to psychological effects, physical disorders, such as high blood pressure, angina complaints, diabetes, and musculoskeletal disorders, may occur by job stress [12].

It was proposed that personality patterns, such as anxiety and depression, as well as stress and negative emotions, can contribute to alterations in the workers’ gastrointestinal tract [13]. Exposure to stress may be a major risk factor for various gastrointestinal tract diseases [14]. A past study also suggested a brain-gut axis, which implies that the brain and gut are linked in such a way that the brain can affect the gut [15]. Mood alteration may be associated with IBS and functional dyspepsia [13].

As shown in the studies reported above, job stress is a risk factor of psychological changes, such as anxiety and depression, which are related to gastrointestinal diseases and symptoms, including functional dyspepsia. However, there are few studies conducted on the relationship between job stress and functional dyspepsia, especially in South Korea.

This study investigated the relationship between job stress and functional dyspepsia among display manufacturing sector workers in South Korea.

## Methods

### Subjects

This study was conducted at a university hospital in Cheonan, Chungcheongnam-do, South Korea from May 1 to July 31, 2016. During this period, employees of the display manufacturing company who participated in health check-ups and agreed to fill out questionnaires were included as study subjects. A total of 901 subjects were enrolled after excluding 196 subjects according to the following exclusion criteria: incomplete assessment questionnaire (*n* = 171) and self-reported organic gastrointestinal disorder (*n* = 25). This study was approved by the institutional review board of Soonchunhyang University Hospital, Cheonan (IRB No. 2017–07–024-001).

### Study procedure

We performed face-to-face interviews using structured questionnaires consisting of questions addressing the subjects’ general characteristics, such as age, alcohol consumption habit, smoking, regular exercise habit, past medical history, sleep disturbance, and job-related details such as duration of shift work, weekly working hours, job stress scale, and symptoms of functional dyspepsia.

Body mass index (BMI), calculated by dividing the measured weight (kg) with the square of height (m^2^), was categorized as ≥25 kg/m^2^ and < 25 kg/m^2^. Alcohol consumption habit was categorized as non-drinker, 1–2 bottles per week, and 3 bottles or more per week. Smoking was categorized as smoker, past smoker, and non-smoker. Regular exercise habit was categorized as exercise 3 times or more every week, exercise 1–2 times every week, and do not exercise. We asked subjects about their past medical history, and those with organic gastrointestinal disorder were excluded from this study.

The Korean occupational stress scale (KOSS) proposed by Chang et al. in 2005 and used to assess job stress for the South Korean population, was used to assess the job stress scale in this study [16]. KOSS was developed and validated by the National Study for Development and Standardization of Occupational Stress [16, 17]. In a study that investigated the reliability and validity of KOSS in occupational therapists, the internal consistency of this tool showed good reliability and questions are also valid [18]. KOSS consists of 8 subcategories and 43 items. The subcategory items are as follows: (1) Physical environment (3 items); (2) Job demand (8 items); (3) Insufficient job control (5 items); (4) Interpersonal conflict (4 items); (5) Job insecurity (6 items); (6) Organizational system (7 items); (7) Lack of reward (6 items); (8) Occupational climate (4 items); Items were scored using 4-point Likert scale for the response categories. The score of each subcategory of KOSS was converted into units 100 point scale. Total sum of all the converted scores in all categories becomes the total job stress score, which is then divided by the number of categories. The Korean Occupational Safety & Health Agency Guide H-67-2012 was used to measure the KOSS score. It is guideline for measuring job stressors, which describes meaning of job stressors and how to use KOSS for workers and health managers in companies. We classified subjects into high-risk group if the KOSS scores were higher than the top 25% Korean workers’ scores (75 percentile) based on the KOSS reference value and low-risk group if the KOSS scores were less than the top 25%.

We surveyed for the duration of shift work and the weekly working hours of subjects. Duration of shift work was classified as less than 5 years, 5–9 years, 10–14 years, and 15 years or more. Weekly working hours were divided into ≤40 h, 41–51 h, and ≥ 52 h. The Korean version of insomnia severity index (ISI-K) was used to assess for sleep disturbance. This is a brief instrument for evaluating the severity of insomnia, and this has been shown to be reliable and valid in the Korean population [19]. It consists of 7 questions described as follows: (1) Difficulty in falling asleep; (2) Difficulty staying asleep; (3) Problems on waking up too early; (4) How satisfied are you with your current sleep pattern?; (5) How noticeable to others do you think your sleep problem is in terms of impairing the quality of your life?; (6) How worried are you about your current sleep problem?; (7) To what extent do you consider your sleep problem to interfere with your daily functioning currently?; These questions used a 5-point Likert scale, and each question scored 0–4 (e.g., 0 = no problem; 4 = very severe problem). Total sum of these 7 scores becomes the total score ranging from 0 to 28. The appropriate cut-off value was reported to be 15.5 points; therefore, we classified subjects with ≥16 points as clinically significant insomnia [19].

The functional dyspepsia component of Rome III diagnostic questionnaire (Rome Foundation) was utilized to evaluate the symptoms of functional dyspepsia. We used the Korean version of this questionnaire to evaluate functional dyspepsia in Korean population [20]. The questionnaire includes the following: postprandial fullness, early satiation, and epigastric pain or burning [2, 20]. Subjects were categorized as functional dyspepsia-positive group (FD-positive group), if they fulfill the following criteria. Firstly, at least 6 months, such subjects have had one or more of either: (1) postprandial fullness or early satiation twice a week or (2) epigastric pain or burning once a week. Secondly, the absence of organic gastrointestinal disorder was confirmed based on the upper gastrointestinal endoscopy findings in the last 2 years. In the absence of endoscopy findings in the last 2 years, subjects underwent upper gastrointestinal endoscopy. Subjects who failed to fulfill these criteria were categorized as functional dyspepsia-negative group (FD-negative group).

### Statistical analysis

Through descriptive statistics and frequency analysis, the general characteristics of subjects and the level of job stress were analyzed. Pearson’s chi-square test and linear-by-linear association test were used to investigate the difference in general characteristics between men and women. T-test was used to determine whether the difference in KOSS scores between men and women was significant. After dividing into groups according to the presence of functional dyspepsia, the general characteristics were analyzed using chi-square test. In order to investigate the relationship between functional dyspepsia and subcategories of KOSS, with the level of job stress as an independent variable and the presence of functional dyspepsia as the dependent variable, we performed multiple logistic regression analysis. We conducted stratified analysis by gender to investigate the difference between men and women. We adjusted for age, BMI, alcohol consumption, regular exercise, smoking, duration of shift work, working hours per week, and ISI-K score in this analysis. Results were presented as odds ratio (OR) and 95% confidence interval (CI). Statistical analysis was performed using SPSS version 19.0 (SPSS, Inc., Chicago, IL, USA) program, and *p*-values < 0.05 were considered statistically significant.

## Results

### Characteristics of study subjects

Men aged 31–35 years were the largest (56.5%) group, followed by 36–40 years (28.9%). Women aged 26–30 years were the largest (35.0%) group, followed by 21–25 years (33.8%). The mean age was 33.11 ± 4.39 years for men and 25.89 ± 4.42 years for women. The proportion of BMI ≥25 kg/m^2^ were higher in men than in women. The proportions of subjects who did not exercise, smoke, or drink alcohol were higher in women than in men. The ratio of non-shift workers was 5.0% in men and 9.8% in women. As the duration of shift work increased, the proportion of men was increased. Differences in age, BMI, regular exercise habit, smoking, alcohol consumption habit, and duration of shift work were significant by Pearson’s chi-square test or linear-by-linear association. However, there was no significant difference in weekly working hours and ISI-K index (Table 1).

**Table 1:** General characteristics of study subjects (*n* = 901)

	Male (%) (*n* = 561)	Female (%) (*n* = 340)	*p*-value
Age (years)			
< 20	24 (4.3%)	53 (15.6%)	< 0.001^*§^
21–25	5 (0.9%)	115 (33.8%)	
26–30	53 (9.4%)	119 (35.0%)	
31–35	317 (56.5%)	45 (13.2%)	
36–40	162 (28.9%)	8 (2.4%)	
Body mass index			
< 25 kg/m^2^	309 (55.1%)	276 (81.2%)	< 0.001^*^
≥ 25 kg/m^2^	252 (44.9%)	64 (18.8%)	
Regular exercise (times / week)			
0	204 (36.4%)	220 (64.7%)	< 0.001^*§^
1–2	239 (42.6%)	89 (26.2%)	
≥ 3	118 (21.0%)	31 (9.1%)	
Smoking			
Non-smoker	196 (34.9%)	285 (83.8%)	< 0.001^*§^
Past smoker	132 (23.5%)	31 (9.1%)	
Current smoker	233 (41.5%)	24 (7.1%)	
Alcohol consumption (times / week)			
0	107 (19.1%)	126 (37.1%)	< 0.001^*§^
1–2	333 (59.4%)	179 (52.6%)	
≥ 3	121 (21.6%)	35 (10.3%)	
Duration of shift work (years)			
< 5	44 (7.8%)	79 (23.2%)	< 0.001^*§^
5–9	190 (33.9%)	152 (44.7%)	
10–14	267 (47.6%)	101 (29.7%)	
≥ 15	60 (10.7%)	8 (2.4%)	
Weekly working hours (hours / week)			
≤ 40	83 (14.8%)	36 (10.6%)	0.491^§^
41–51	383 (68.3%)	252 (74.1%)	
≥ 52	95 (16.9%)	52 (15.3%)	
ISI-K index (score)			
Non-significant insomnia (0–15)	530 (94.5%)	312 (91.8%)	0.111
Clinically significant insomnia (16–28)	31 (5.5%)	28 (8.2%)	
Functional dyspepsia			
(−)	533 (95.0%)	311 (91.5%)	0.034^*^
(+)	28 (5.0%)	29 (8.5%)	

*ISI-K* Korean version of insomnia severity index

^§^*p*-value of linear by linear association

^*^p < 0.05

### Prevalence of functional dyspepsia and job stress level of study subjects

The Pearson’s chi-square test was conducted to identify differences between FD-positive and FD-negative groups by sex. The proportion of FD-positive group was higher in women (8.5%) than men (5.0%) (Table 1). The total job stress score of KOSS by sex was 55.43 ± 9.04 in men and 58.27 ± 7.65 in women (data not shown), and this difference was significant (Table 2). In job stress score by subcategories, job insecurity, job demand, insufficient job control, organizational system, and lack of reward scores were higher in women than men; these differences were significant. Comparing the KOSS reference score for high-risk group with the median for each subcategory, the median values of insufficient job control, job insecurity and organizational system were higher than the reference value in men. The median values of insufficient job control, interpersonal conflict, job insecurity, organizational system, and total job stress score were higher than the reference value in women. Those categorized into high-risk groups by the total job stress score were 239 (42.6%) men and 202 (59.4%) women.

**Table 2:** Job stress levels of study subjects (*n* = 901)

	Male (*n* = 561)				Female (*n* = 340)			
	Mean ± SD	Median	Reference^a^	Subjects of high risk (%)	Mean ± SD	Median	Reference^a^	Subjects of high risk (%)
Physical environment	46.96 ± 13.65	41.80	66.70	54 (9.6%)	45.25 ± 13.69	41.80	55.60	84 (24.7%)
Job demand	42.73 ± 12.27^*^	40.30	58.40	75 (13.4%)	47.43 ± 13.60^*^	45.20	62.60	39 (11.5%)
Insufficient job control	71.33 ± 10.29^*^	70.00	60.10	464 (82.7%)	80.23 ± 9.02^*^	80.30	66.70	312 (91.8%)
Interpersonal conflict	54.76 ± 15.40	50.00	50.10	280 (49.9%)	55.87 ± 15.92	56.10	41.70	278 (81.8%)
Job insecurity	62.51 ± 10.90^*^	62.40	61.20	332 (59.2%)	59.68 ± 10.90^*^	58.35	55.60	213 (62.6%)
Organizational system	66.87 ± 15.30^*^	67.90	62.00	347 (61.9%)	71.14 ± 13.10^*^	71.50	62.00	248 (72.9%)
Lack of reward	59.05 ± 14.37^*^	58.20	77.80	65 (11.6%)	67.18 ± 12.79^*^	66.40	77.80	82 (24.1%)
Occupational climate	39.20 ± 14.95	37.30	50.10	91 (16.2%)	39.38 ± 13.41	37.30	50.10	63 (18.5%)
Total job stress score	55.43 ± 9.04^*^	54.95	56.60	239 (42.6%)	58.27 ± 7.65^*^	57.98	56.70	202 (59.4%)

*SD* standard deviation

^*^Statistical significant difference by t-test (*p* < 0.05)

^a^KOSS reference value (75 percentile)

### Functional dyspepsia according to characteristics

We performed the chi-square test to compare FD-positive with FD-negative groups by general characteristics and other features. There were more FD-positive group subjects with ISI-K index ≥16, and the difference was significant in both men (*p* < 0.001) and women (*p* = 0.011). There were no significant differences with other characteristics or features (Table 3).

**Table 3:** Distribution of workers with functional dyspepsia by characteristics (*n* = 901)

	Male (*n* = 561)			Female (*n* = 340)		
	FD(−) N	FD(+) N	*p*-value	FD(−) N	FD(+) N	*p*-value
Age (years)						
< 20	22 (91.7%)	2 (8.3%)	0.456	48 (90.6%)	5 (9.4%)	0.967
21–25	5 (100%)	0 (0%)		106 (92.2%)	9 (7.8%)	
26–30	48 (90.6%)	5 (9.4%)		108 (90.8%)	11 (9.2%)	
31–35	302 (95.3%)	15 (4.7%)		42 (93.3%)	3 (6.7%)	
36–40	156 (96.3%)	6 (3.7%)		7 (87.5%)	1 (12.5%)	
Body mass index						
< 25 kg/m^2^	297 (96.1%)	12 (3.9%)	0.182	252 (91.3%)	24 (8.7%)	0.820
≥ 25 kg/m^2^	236 (93.7%)	16 (6.3%)		59 (92.2%)	5 (7.8%)	
Regular exercise (times / week)						
0	194 (95.1%)	10 (4.9%)	0.997	203 (92.3%)	17 (7.7%)	0.617
1–2	227 (95.0%)	12 (5.0%)		81 (91.0%)	8 (9.0%)	
≥ 3	112 (94.9%)	6 (5.1%)		27 (87.1%)	4 (12.9%)	
Smoking						
Non-smoker	184 (93.9%)	12 (6.1%)	0.662	262 (91.9%)	23 (8.1%)	0.228
Past smoker	126 (95.5%)	6 (4.5%)		26 (86.7%)	5 (16.1%)	
Current smoker	223 (95.7%)	10 (4.3%)		23 (95.8%)	1 (4.2%)	
Alcohol consumption (times / week)						
0	104 (97.2%)	3 (2.8%)	0.506	116 (92.1%)	10 (7.9%)	0.362
1–2	315 (94.6%)	18 (5.4%)		161 (89.9%)	18 (10.1%)	
≥ 3	114 (94.2%)	7 (5.8%)		34 (97.1%)	1 (2.9%)	
Duration of Shift work (years)						
< 5	42 (95.5%)	2 (4.5%)	0.956^#^	72 (91.1%)	7 (8.9%)	0.863
5–9	179 (94.2%)	11 (5.8%)		141 (92.8%)	11 (7.2%)	
10–14	254 (95.1%)	13 (4.9%)		91 (90.1%)	10 (9.9%)	
≥ 15	58 (96.7%)	2 (3.3%)		7 (87.5%)	1 (12.5%)	
Weekly working hours (hours / week)						
≤ 40	81 (97.6%)	2 (2.4%)	0.444	34 (94.4%)	2 (5.6%)	0.148
41–51	363 (94.8%)	20 (5.2%)		233 (92.5%)	19 (7.5%)	
≥ 52	89 (93.7%)	6 (6.3%)		44 (84.6%)	8 (15.4%)	
ISI-K index (score)						
Non-significant insomnia (0–15)	509 (96.0%)	21 (4.0%)	< 0.001^*^	289 (92.6%)	23 (7.4%)	0.011^*^
Clinically significant insomnia (16–28)	24 (77.4%)	7 (22.6%)		22 (78.6%)	6 (21.4%)	

*FD(−) N* the number of functional dyspepsia-negative group, *FD(+) N* the number of functional dyspepsia-positive group, *ISI-K* Korean version of insomnia severity index

*p*-value: *p*-value of chi-square tests; ^*^p < 0.05; ^#^*p*-value of fisher’s exact test

### Job stress factors and functional dyspepsia

Logistic regression analysis was performed to investigate whether the occurrence of functional dyspepsia correlated with the total job stress score and the 8 subcategory scores of KOSS. Subjects were divided into the high- and low-risk groups in each of the 8 subcategories of KOSS. We built two models, Model I was non-adjusted while Model II was adjusted for age, BMI, alcohol consumption, regular exercise, smoking, duration of shift work, working hours per week, and ISI-K score. There were no statistically significant correlations in men. However, in women, the risk of functional dyspepsia was significantly higher in the high-risk groups of the following KOSS subcategories in Model I: job demand (OR 3.282, 95% CI 1.181–9.126), and occupational climate (OR 2.665, 95% CI 1.041–6.823). Even after adjustment, in Model II, the risk was significantly higher in the high-risk groups of the following KOSS subcategories: job demand (OR 3.123, 95% CI 1.036–9.416) and occupational climate (OR 3.304, 95% CI 1.198–9.115) (Table 4).

**Table 4:** Odds ratios and 95% confidence intervals for functional dyspepsia and job stress (*n* = 901)

	Male (*n* = 561)				Female (*n* = 340)			
	Model I		Model II		Model I		Model II	
	OR	95% CI	OR	95% CI	OR	95% CI	OR	95% CI
Physical environment								
Low risk	1		1		1		1	
High risk	1.501	0.467–4.817	1.377	0.407–4.656	1.002	0.386–2.604	1.239	0.434–3.541
Job demand								
Low risk	1		1		1		1	
High risk	1.344	0.451–4.004	1.039	0.308–3.500	3.282^*^	1.181–9.126	3.303^*^	1.056–10.328
Insufficient job control								
Low risk	1		1		1		1	
High risk	1.484	0.418–5.264	1.294	0.339–4.949	3.408	0.416–27.922	4.165	0.382–45.452
Interpersonal conflict								
Low risk	1		1		1		1	
High risk	2.150	0.820–5.637	2.076	0.736–5.854	1.735	0.459–6.557	2.296	0.540–9.755
Job insecurity								
Low risk	1		1		1		1	
High risk	0.566	0.230–1.390	0.762	0.289–2.009	1.048	0.432–2.540	1.246	0.465–3.337
Organizational system								
Low risk	1		1		1		1	
High risk	0.742	0.242–2.277	0.570	0.164–1.975	1.294	0.439–3.817	1.449	0.446–4.712
Lack of reward								
Low risk	1		1		1		1	
High risk	0.482	0.128–1.812	0.457	0.109–1.912	0.823	0.308–2.196	0.829	0.293–2.344
Occupational climate								
Low risk	1		1		1		1	
High risk	2.077	0.767–5.629	1.888	0.657–5.428	2.665^*^	1.041–6.823	3.795^*^	1.303–11.056
Total job stress score								
Low risk	1		1		1		1	
High risk	1.418	0.395–5.086	1.897	0.471–7.636	0.631	0.191–2.084	0.421	0.116–1.532

Model I: Unadjusted; Model II: Adjusted (age, BMI, alcohol consumption, regular exercise, smoking, duration of shift work, working hours per week, ISI-K score)

*OR* odds ratio, *CI* confidence interval

^*^p < 0.05

## Discussion

This study investigated the relationship between job stress and functional dyspepsia in display manufacturing sector workers. Even after adjusting for variables, the prevalence of functional dyspepsia was higher in the job demand and occupational climate high-risk groups, with statistical significance in women.

There are three systems related to the mechanism by which stress has been proposed to produce gastrointestinal tract alterations in workers: the sympathetic autonomic nervous system (ANS), the hypothalamus-pituitary-adrenal axis, and genetic factors [13]. The ANS regulates gastrointestinal tract motility by controlling the peristaltic activity through the myenteric system [21, 22]. A relationship between stress and delayed gastric emptying or other motor disturbances was suggested in a previous study [23]. When attempting to achieve both physical and psychological balance, the human body reacts defensively. However, the activation of adaptive or allostatic systems can become maladaptive because of frequent, chronic, or excessive stress; thereby, predisposing to disease [15]. The concept of the brain-gut interaction was suggested from this explanation [24]. It has been hypothesized that exposure to psychological stress causes alterations in the brain-gut interactions, ultimately leading to the development of a broad array of gastrointestinal tract disorders, including functional gastrointestinal disorders. The neuroendocrine response to stress is mediated by the corticotrophin-releasing hormone (CRH). In the brain-gut axis, CRH is considered a major mediator of the stress response [13]. The stress-related activation of CRH receptors has been reported to produce alterations in gastrointestinal function. Central or peripheral administration of CRH can produce accelerated colonic motor function and can be blocked by treatment with a variety of CRH antagonists [25].

In a meta-analysis and a community study, an association was shown between anxiety, depression, and functional dyspepsia [5, 26], and this has been confirmed in many studies published thereafter [27]. A higher prevalence of physical and emotional abuse was reported in patients with functional dyspepsia than in healthy controls in adulthood [28]. Furthermore, a Norwegian study showed that patients with functional dyspepsia experienced significantly more stressful life events than patients with duodenal ulcer or healthy controls [29].

Job stress has become one of the most serious health issues in the modern world [30]. The concept of job stress has been observed to be a natural extension of the classical concept of stress [13]. Job stress is related to psychological stress, and may affect different physiological functions in the gastrointestinal tract [31]. However, evaluation of stress factors are the most complicated factors to be studied quantitatively.

Previous studies suggest that higher job stress in job demand and occupational climate has a relationship with gastrointestinal alterations. The findings of our study agree with those of previous studies in terms of context. By investigating display manufacturing sector workers and the relationship between functional dyspepsia and KOSS subcategories, the present study has broadened our scope and understanding of psychosocial factors in functional dyspepsia.

There are several previous studies that found association between evaluated job stress factors and gastrointestinal symptoms. A German study investigated an association between job-related psychosocial factors and the occurrence of dyspepsia symptoms in white collar employees. This study showed that dyspepsia symptom score was significantly higher in employees who had a critical style of coping with work demands (OR 3.22, 95% CI 1.56–6.65) [32]. Another study examined the relationship between job stress and gastric disease among shipbuilding male workers. It was reported that the risk of gastric disease was significantly higher in the high job stress group than in the low job stress group in the occupational climate subcategory (OR 2.82, 95% CI 1.15–6.91) [33]. In a study on the effects of psychosocial factors of IBS among firefighters, the risk of IBS was higher in the following KOSS subcategories: job demand (OR 1.79, 95% CI 1.11–2.89), interpersonal conflict (OR 2.21, 95% CI 1.25–4.33), and lack of reward (OR 2.39, 95% CI 1.08–5.26) [34].

In the current study, the association between job stress and functional dyspepsia was not significant in men, but in women. Several studies have reported that women can be more affected by stress than men. A British study emphasized that emotional and environmental states in women play an important role in the development of IBS [24]. it has been proven that women present with the highest prevalence of physical and psychological symptoms compared with males worldwide [35]. In a study in Japan, it was found that women are more likely to experience occupational stress than men [36]. Recent studies showed the importance of gender on stress and revealed that women report higher levels of chronic and daily stressors than men [37]. These findings could affect the results in this study. It is difficult to state with certainty that women are more likely to experience stress because this present study was cross-sectional in design; however, it is possible that the emotional and environmental states and style of coping with stress in women might have affected our results.

This study has some limitations. First, it was a cross-sectional study; therefore, it was hard to clearly identify a causal relationship. Second, because we conducted the investigation among manufacturing sector workers, a specific occupational group, it is difficult to generalize the results to workers in other jobs. Therefore, in future studies, workers in different jobs should be selected to ensure inclusion of the different exposures to work content, environment, and different types or levels of job stress. Third, because of the characteristics of manufacturing sector workers, most of the subjects were shift workers. Thus, it may be difficult to apply the results of this study to non-shift workers. Fourth, there are prior studies which examined associations between dyspepsia and basic socio-demographic features such as education, income, marital status [38, 39]. However, this study could not investigate these associations because we had not included these features in our survey. Thus, future studies are needed to include these features in investigation. Fifth, the KOSS reference value may be not a perfect cut-off score, because it was established in 13 years ago [16], and subjects in this study were young people aged 40 years or less.

Nonetheless, this study was conducted with a large number of subjects. Furthermore, the study also offered the advantage of examining the influence of job stress on functional dyspepsia in the manufacturing sector; which plays a critical role in employment in South Korea by employing over 3.5 million workers. This study provides findings of statistical analysis disaggregated by sex. This suggests differences in the prevalence of functional dyspepsia and the effects of job stress factors among male and female workers, which have hardly been investigated previously.

## Conclusions

In conclusion, job demand and occupational climates were associated with functional dyspepsia in female manufacturing sector workers. Therefore, both clinical and mental health approaches should be used in the management of functional dyspepsia in women. Additionally, more interest and further research into mental health and job stress of those employed in jobs, including manufacturing sector workers along with other jobs, is necessary.

## Abbreviations

ANS: Autonomic nervous system
BMI: Body mass index
CI: Confidence interval
CRH: Corticotrophin-releasing hormone
FD: Functional dyspepsia
IBS: Irritable bowel syndrome
ISI-K: Korean version of insomnia severity index
KOSS: Korean Occupational Stress Scale
OR: Odds ratio
SD: Standard deviation

## References

[1] Drossman DA (2006). The functional gastrointestinal disorders and the Rome III process. Gastroenterology.

[2] Tack J, Talley NJ, Camilleri M, Holtmann G, Hu P, Malagelada JR (2006). Functional gastroduodenal disorders. Gastroenterology.

[3] Lee H, Jung H-K, Huh KC (2014). Current status of functional dyspepsia in Korea. Korean J Intern Med.

[4] Miwa H, Kusano M, Arisawa T, Oshima T, Kato M, Joh T (2015). Evidence-based clinical practice guidelines for functional dyspepsia. J Gastroenterol.

[5] Henningsen P, Zimmermann T, Sattel H (2003). Medically unexplained physical symptoms, anxiety, and depression: a meta-analytic review. Psychosom Med.

[6] Haug TT, Mykletun A, Dahl AA (2002). Are anxiety and depression related to gastrointestinal symptoms in the general population?. Scand J Gastroenterol.

[7] Sauter SM, Murphy L, Colligan M, Swanson N, Hurrell J, Scharf F (1999). STRESS...At Work. US National Institute for Occupational Safety and Health, DHHS (NIOSH) Publication.

[8] Chang SJ, Koh SB, Kang MG, Cha BS, Park JK, Hyun SJ (2005). Epidemiology of psychosocial distress in Korean employees. J Prev Med Public Health.

[9] Huston M, Larsen RC, LaDou J, Harrison RJ (2014). Occupational Mental Health & Workplace Violence. Current Diagnosis & Treatment: occupational and environmental medicine.

[10] Melchior M, Caspi A, Milne BJ, Danese A, Poulton R, Moffitt TE (2007). Work stress precipitates depression and anxiety in young, working women and men. Psychol Med.

[11] Park KC, Lee KJ, Park JB, Min KB, Lee KW (2008). Association between occupational stress and depression, anxiety, and stress symptoms among white-collar male workers in an automotive company. Korean J Occup Environ Med.

[12] Houtman I, Jettinghoff K, Cedillo L. Protecting Workers' Health Series No. 6: Raising awareness of stress at work in developing countries. Geneva: WHO Press; 2007. http://www.who.int/occupational_health/publications/pwh6/en/. Accessed 28 Mar 2018.

[13] Huerta-Franco M-R, Vargas-Luna M, Tienda P, Delgadillo-Holtfort I, Balleza-Ordaz M, Flores-Hernandez C (2013). Effects of occupational stress on the gastrointestinal tract. World J Gastrointest Pathophysiol.

[14] Konturek PC, Brzozowski T, Konturek SJ (2011). Stress and the gut: pathophysiology, clinical consequences, diagnostic approach and treatment options. J Physiol Pharmacol.

[15] Bhatia V, Tandon RK (2005). Stress and the gastrointestinal tract. J Gastroenterol Hepatol.

[16] Chang SJ, Koh SB, Kang D, Kim SA, Kang MG, Lee CG (2005). Developing an occupational stress scale for Korean employees. Korean J Occup Environ Med..

[17] Kang SH, Boo YJ, Lee JS, Ji WB, Yoo BE, You JY (2013). Analysis of the occupational stress of Korean surgeons: a pilot study. J Korean Surg Soc.

[18] Choi YI, Kim EJ, Park EY (2011). Validity of the Korean occupational stress scale in occupational therapists. J Korea Contents Association.

[19] Cho YW, Song ML, Morin CM (2014). Validation of a Korean version of the insomnia severity index. J Clin Neurol.

[20] Song KH, Jung HK, Min BH, Youn YH, Choi KD, Keum BR (2013). Development and validation of the Korean Rome III questionnaire for diagnosis of functional gastrointestinal disorders. J Neurogastroenterol Motil.

[21] Sobreira LF, Zucoloto S, Garcia SB, Troncon LE (2002). Effects of myenteric denervation on gastric epithelial cells and gastric emptying. Dig Dis Sci.

[22] Quintana E, Hernandez C, Alvarez-Barrientos A, Esplugues JV, Barrachina MD (2004). Synthesis of nitric oxide in postganglionic myenteric neurons during endotoxemia: implications for gastric motor function in rats. FASEB J.

[23] Quigley EM (2004). Review article: gastric emptying in functional gastrointestinal disorders. Aliment Pharmacol Ther.

[24] Mawdsley JE, Rampton DS (2005). Psychological stress in IBD: new insights into pathogenic and therapeutic implications. Gut.

[25] Sagami Y, Shimada Y, Tayama J, Nomura T, Satake M, Endo Y (2004). Effect of a corticotropin releasing hormone receptor antagonist on colonic sensory and motor function in patients with irritable bowel syndrome. Gut.

[26] Mak A. D. P., Wu J. C. Y., Chan Y., Chan F. K. L., Sung J. J. Y., Lee S. (2012). Dyspepsia is strongly associated with major depression and generalised anxiety disorder - a community study. Alimentary Pharmacology & Therapeutics.

[27] Van Oudenhove L, Aziz Q (2013). The role of psychosocial factors and psychiatric disorders in functional dyspepsia. Nat Rev Gastroenterol Hepatol.

[28] Koloski NA, Talley NJ, Boyce PM (2005). A history of abuse in community subjects with irritable bowel syndrome and functional dyspepsia: the role of other psychosocial variables. Digestion.

[29] Haug TT, Wilhelmsen I, Berstad A, Ursin H (1995). Life events and stress in patients with functional dyspepsia compared with patients with duodenal ulcer and healthy controls. Scand J Gastroenterol.

[30] Chang Boon Lee P (2003). Going beyond career plateau: using professional plateau to account for work outcomes. J Manag Dev.

[31] Huerta-Franco MR, Vargas-Luna M, Montes-Frausto JB, Morales-Mata I, Ramirez-Padilla L (2012). Effect of psychological stress on gastric motility assessed by electrical bio-impedance. World J Gastroenterol.

[32] Rothenbacher D, Peter R, Bode G, Adler G, Brenner H (1998). Dyspepsia in relation to helicobacter pylori infection and psychosocial work stress in white collar employees. Am J Gastroenterol.

[33] Lihm HS, Park SH, Gong EH, Choi JS, Kim JW (2012). Relationship between occupational stress and gastric disease in male workers. Korean J Fam Med.

[34] Jang SH, Ryu HS, Choi SC, Lee SY (2017). Psychological factors influence the irritable bowel syndrome and their effect on quality of life among firefighters in South Korea. Psychiatry Investig.

[35] Huerta R, Brizuela-Gamino OL (2002). Interaction of pubertal status, mood and self-esteem in adolescent girls. J Reprod Med.

[36] Nakadaira H, Yamamoto M, Matsubara T (2006). Mental and physical effects of Tanshin funin, posting without family, on married male workers in Japan. J Occup Health.

[37] Gentry LA, Chung J, Aung N, Keller S, Heinrich KM, Maddock J (2007). Gender differences in stress and coping among adults living in Hawaii. Calif J Health Promot.

[38] Drossman DA, Li Z, Andruzzi E, Temple RD, Talley NJ, Thompson WG (1993). U.S. householder survey of functional gastrointestinal disorders. Prevalence, sociodemography, and health impact. Dig Dis Sci.

[39] Tougas G, Chen Y, Hwang P, Liu MM, Eggleston A (1999). Prevalence and impact of upper gastrointestinal symptoms in the Canadian population: findings from the DIGEST study. Domestic/International Gastroenterology Surveillance Study. Am J Gastroenterol.

